# Valor Diagnóstico dos Níveis Séricos de sCD40L, CCL3 e NT-proBNP na Detecção da Trombose Venosa de Membros Inferiores em Pacientes Idosos com Insuficiência Cardíaca

**DOI:** 10.36660/abc.20250445

**Published:** 2026-03-02

**Authors:** Xuelian Liu, Lina Mu, Shasha Ma, Huijing Zhang

**Affiliations:** 1 Harrison International Peace Hospital Department of Cardiovascular Medicine Hengshui China Department of Cardiovascular Medicine, Harrison International Peace Hospital, Hengshui – China; 2 Hengshui Sixth People's Hospital Department of Surgery Hengshui China Department of Surgery, Hengshui Sixth People's Hospital, Hengshui – China

**Keywords:** Trombose Venosa, Insuficiência Cardíaca, Idoso

## Abstract

**Fundamento:**

Os níveis séricos do ligante solúvel de CD40 (sCD40L), da quimiocina ligante 3 (CCL3) e do peptídeo natriurético cerebral N-terminal pró-hormonal (NT-proBNP) estão intimamente associados à insuficiência cardíaca (IC).

**Objetivo:**

Este estudo avaliou o valor preditivo dos níveis séricos de sCD40L, CCL3 e NT-proBNP para trombose venosa de membros inferiores (TVMI) em pacientes idosos com IC.

**Métodos:**

Um total de 200 pacientes com IC foi incluído retrospectivamente e igualmente dividido em grupos com trombo e sem trombo, com base nos achados do ultrassom de veias dos membros inferiores. Os níveis séricos de sCD40L, CCL3 e NT-proBNP foram medidos e analisados. Um valor de p < 0,05 foi considerado estatisticamente significativo.

**Resultados:**

Os níveis séricos de sCD40L, CCL3 e NT-proBNP foram significativamente mais elevados no grupo com trombo em comparação com o grupo sem trombo (p < 0,05). A análise de regressão logística multivariada demonstrou que níveis séricos elevados de sCD40L, CCL3 e NT-proBNP são fatores de risco para TVMI em pacientes com IC (p < 0,05). A área sob a curva característica de operação do receptor (ROC) (AUC) para os níveis séricos combinados de sCD40L, CCL3 e NT-proBNP foi maior do que a de cada marcador individual (valores de Z: sCD40L = 3,337; CCL3 = 2,303; e NT-proBNP = 5,156; p < 0,001; p < 0,001; e p = 0,021, respectivamente).

**Conclusão:**

Níveis séricos elevados de sCD40L, CCL3 e NT-proBNP foram associados à TVMI em pacientes idosos com IC. A combinação desses marcadores oferece alto valor diagnóstico para a detecção de TVMI nessa população.

**Figure:**
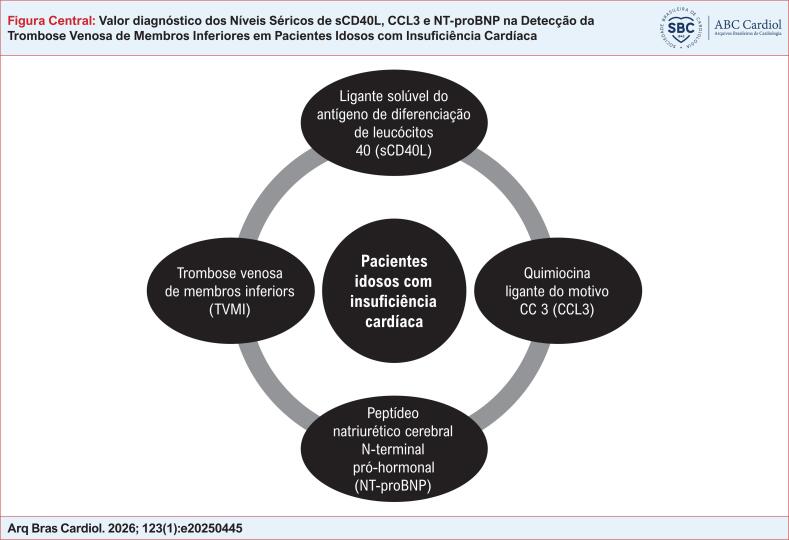
1. Os níveis séricos de sCD40L, CCL3 e NT-proBNP foram significativamente mais elevados no grupo com trombo em comparação com o grupo sem trombo; 2. Níveis séricos elevados de sCD40L, CCL3 e NT-proBNP representam fatores de risco para TVMI em pacientes com Insuficiência Cardíaca (IC); 3. A área sob a curva característica de operação do receptor (ROC) para os níveis séricos combinados de sCD40L, CCL3 e NT-proBNP foi maior do que a de cada marcador individual.

## Introdução

A insuficiência cardíaca (IC) é uma condição clínica multifatorial que resulta de defeitos estruturais ou funcionais do coração, limitando sua capacidade de contrair ou de os ventrículos relaxarem. A incidência de IC na China tem aumentado de forma constante em paralelo ao envelhecimento populacional.^1,2^ Pacientes com IC geralmente apresentam sintomas como dispneia, fadiga e retenção de líquidos.^1,2^

A trombose venosa de membros inferiores (TVMI) é um distúrbio concomitante comum em pacientes com IC. O Tromboembolismo Venoso (TEV), que inclui tanto a Embolia Pulmonar (EP) quanto a Trombose Venosa Profunda (TVP/TVMI), representa um risco particularmente grave para indivíduos com IC. De fato, pacientes com IC têm um risco 2–3 vezes maior de desenvolver TVMI e TEV em comparação à população geral.^3,4^ Esse risco aumentado é amplamente atribuído a fatores como imobilidade, infecções agudas e comorbidades, incluindo a Doença Pulmonar Obstrutiva Crônica (DPOC), que por si só eleva o risco de TEV.^5–7^ Pacientes com IC podem apresentar consequências graves da TVP, incluindo insuficiência ventricular direita e aumento da mortalidade. A redução da velocidade do fluxo sanguíneo nos membros inferiores devido à TVP leva à inflamação da parede venosa e a um estado de hipercoagulabilidade, que compromete o retorno venoso. O risco de mortalidade pode aumentar se o trombo se desprender e causar uma embolia pulmonar.^8,9^ Portanto, o diagnóstico preciso e a intervenção oportuna em pacientes com IC e TVMI são essenciais para melhorar o prognóstico.

Uma TVP em pacientes com IC está associada a fatores de risco, como idade avançada, sexo feminino e histórico de TVP ou EP. Os fatores de risco incluem anormalidades hereditárias da coagulação, doenças crônicas (como câncer), obesidade [Índice de Massa Corporal (IMC) elevado] e imobilidade prolongada. Fatores adicionais que podem aumentar a vulnerabilidade incluem cateteres venosos centrais, efeitos hormonais (como o estrogênio proveniente de terapia hormonal) e lesões nas veias decorrentes de trauma ou cirurgia. Em conjunto, essas variáveis demonstram a complexa interação entre fatores clínicos e demográficos que influenciam o risco de TVP em pacientes com IC.^10,11^

O grau e o prognóstico da IC estão correlacionados com o biomarcador peptídeo natriurético cerebral N-terminal (NT-ProBNP), que reflete a função cardíaca.^12^ O NT-ProBNP é essencial para o diagnóstico e avaliação da IC. Estudos demonstraram que fatores inflamatórios desempenham um papel importante na formação da TVP.^13^ Para regular as respostas inflamatórias, as plaquetas liberam o ligante solúvel do antígeno de diferenciação leucocitária 40 (sCD40L), uma glicoproteína transmembrana pertencente à família dos receptores do fator de necrose tumoral.^14^ Jin et al.^15^ relataram que pacientes idosos com IC grave apresentam níveis séricos elevados de sCD40L. A interação entre CD40 e sCD40L é crítica em condições inflamatórias. Os níveis de sCD40L são significativamente maiores em pacientes com IC, especialmente naqueles com IC grave, diabetes ou hipertensão, em comparação com controles saudáveis. O aumento do sCD40L pode impactar a função vascular e o remodelamento cardíaco, conforme evidenciado pela correlação com a gravidade clínica, o desequilíbrio neuro-hormonal e a disfunção ventricular esquerda.^16^ Outro estudo mostrou que pacientes com asma brônquica e pacientes com osteoporose apresentam níveis elevados da quimiocina pró-inflamatória CCL3, que desempenha papel importante no controle dos processos inflamatórios.^17^

A ultrassonografia venosa dos membros inferiores é considerada o "padrão-ouro" para o diagnóstico da TVP; entretanto, trata-se de um procedimento de alto custo. Biomarcadores séricos podem ser utilizados clinicamente para identificar índices associados à TVP e podem facilitar o diagnóstico em pacientes idosos com IC. A aplicação diagnóstica dos níveis séricos de sCD40L, CCL3 e NT-proBNP em pacientes com IC e TVP tem sido limitada. O presente estudo avaliou a utilidade diagnóstica dos níveis séricos de sCD40L, CCL3 e NT-proBNP em 200 pacientes idosos com IC, com o objetivo de fornecer subsídios para o diagnóstico clínico da IC concomitante à TVP.

## Materiais e métodos

Um total de 200 pacientes idosos com IC, que receberam tratamento em nossa instituição entre julho de 2020 e julho de 2022, foi incluído neste estudo. Os pacientes foram categorizados em dois grupos (trombo e não trombo) com base nos resultados da ultrassonografia venosa dos membros inferiores no momento da admissão. O estudo seguiu os princípios éticos e os padrões para experimentação em seres humanos e foi aprovado pelo Comitê de Ética Médica de nosso hospital (n° 2022-1-035). Cada participante forneceu consentimento informado por escrito.

### Critérios de inclusão

Os critérios de inclusão foram os seguintes: diagnóstico de IC com base no Consenso de Especialistas de 2021 sobre Avaliação da Qualidade e Indicadores de Controle para o Diagnóstico e Tratamento da Insuficiência Cardíaca na China, considerando histórico médico, sintomas, sinais, ecocardiografia e avaliação da função cardíaca;^18,19^ concordância em realizar ultrassonografia venosa dos membros inferiores; idade superior a 60 anos; disponibilidade de dados clínicos completos.

### Critérios de exclusão

Os critérios de exclusão foram os seguintes: presença de varizes nos membros inferiores, neoplasias, doenças hematológicas, EP, diabetes, fibrilação atrial ou DPOC; uso de anticoagulantes (incluindo varfarina, rivaroxabana, dabigatrana ou heparina de baixo peso molecular) antes da admissão; gestação ou lactação; disfunção grave de órgãos vitais, incluindo coração, fígado, baço ou rins; ausência de consentimento informado do paciente ou de sua família.

### Principais reagentes e instrumentos

Os seguintes instrumentos e reagentes foram utilizados neste estudo: Philips Epiq 5C (Philips Healthcare, Cidade, Países Baixos); Analisador Bioquímico Automatizado Hitachi 7600 (Hitachi High-Technologies Corporation, Cidade, Japão); Leitor de Microplacas Multifuncional FK-SY96S (Shandong Lain Optoelectronics Technology Co., Ltd., Cidade, China); e kits de ELISA para sCD40L e CCL3 (Abcam, Cambridge, Reino Unido).

### Coleta de informações gerais

Foram coletados dados de todos os participantes, incluindo idade, sexo, IMC, pressão arterial sistólica, pressão arterial diastólica, glicemia de jejum, Colesterol Total (CT), Triglicerídeos (TG), colesterol de lipoproteína de alta densidade (HDL-C), colesterol de lipoproteína de baixa densidade (LDL-C), lipoproteína (a) [Lp(a)], além do histórico médico de diabetes e hipertensão, juntamente com outros indicadores clínicos.

### Exame de ultrassom

Um técnico realizou exames de ultrassom venoso dos membros inferiores à beira do leito imediatamente após a inclusão dos participantes no estudo, utilizando um aparelho de ultrassom equipado com transdutor linear de alta frequência (faixa de frequência: 6–12 MHz). A avaliação incluiu a veia femoral comum, veia femoral, veia femoral profunda (profunda femoris), veia poplítea, veia tibial anterior, veia tibial posterior, veia fibular (peroneal), veia dorsal do pé, veias intermusculares da perna e veias comunicantes. O diagnóstico de TVMI foi baseado nos critérios descritos na publicação de 2018 *Lower Limb Venous Ultrasound: A Review of the American Institute of Ultrasound in Medicine Guidelines for Peripheral Venous Ultrasound Examination*. Os pacientes foram posteriormente classificados em grupos com trombo ou sem trombo.

### Coleta e armazenamento de soro

Na manhã seguinte à admissão, foram coletados 4 mL de sangue periférico de cada paciente com IC em tubos, que permaneceram em repouso à temperatura ambiente por 30 minutos. Para obtenção do soro, as amostras foram centrifugadas por 3 minutos a 3000 rpm. Em seguida, o soro foi alicotado em tubos estéreis tipo EP e armazenado a −20 °C.

### Mensuração dos níveis séricos de sCD40L, CCL3 e NT-ProBNP

Os níveis séricos de NT-ProBNP foram determinados utilizando um analisador bioquímico automatizado. As concentrações séricas de sCD40L e CCL3 foram medidas de acordo com as instruções do fabricante fornecidas com os respectivos kits de ensaio.

### Análise estatística

As análises estatísticas foram realizadas utilizando o SPSS versão 23.0. As variáveis contínuas são apresentadas como média ± Desvio Padrão (DP), e a normalidade foi avaliada pelo teste de Shapiro–Wilk. As diferenças nas características basais e nos níveis de sCD40L, CCL3 e NT-ProBNP entre os dois grupos foram comparadas utilizando o teste t de Student não pareado. As associações entre os níveis de sCD40L, CCL3 e NT-ProBNP foram avaliadas por meio da análise de correlação de Pearson. A ocorrência de TVMI em pacientes com IC foi investigada por meio de análise de regressão logística. As variáveis independentes incluídas no modelo de regressão logística multivariada foram Lp(a), contagem de plaquetas, creatina quinase, sCD40L, CCL3 e níveis de NT-ProBNP. O desempenho diagnóstico dos níveis séricos de sCD40L, CCL3 e NT-ProBNP para a identificação de trombose venosa em pacientes com IC foi avaliado por meio da análise da curva característica de operação do receptor (ROC). As comparações da área sob a curva ROC (AUC) foram realizadas utilizando o teste Z. Valores de P < 0,05 foram considerados estatisticamente significativos.

## Resultados

Dados clínicos foram coletados de 200 pacientes, que foram divididos em grupo sem trombose (n = 100) e grupo com trombose (n = 100). Não houve diferenças significativas em relação ao sexo e à idade entre os dois grupos (Tabela 1).

**Tabela 1:** Comparação dos dados clínicos gerais entre os grupos sem trombose e com trombose (Média±DP)

Indicadores clínicos	Grupo sem trombose (n=100)	Grupo com trombose (n=100)	p
Idade (anos)	69,12±5,26	68,54±5,84	0,461
Homens/mulheres (casos)	50/50	55/45	0,479
IMC (Kg/m^2^)	22,18±2,69	21,92±3,02	0,521
Pressão arterial sistólica (mmHg)	132,54±17,48	136,20±18,17	0,148
Pressão arterial diastólica (mmHg)	81,25±9,62	83,33±9,34	0,122
Glicemia de jejum (mmol/L)	4,86±0,85	4,93±0,74	0,535
CT (mmol/L)	4,05±0,36	4,09±0,40	0,458
TG (mmol/L)	2,26±0,18	2,31±0,20	0,065
HDL-C (mmol/L)	1,89±0,52	1,95±0,47	0,393
LDL-C (mmol/L)	2,65±0,30	2,68±0,32	0,495
Lp(a) (mg/L)	187,56±26,28	332,04±37,41	<0,001
História de diabetes mellitus (sim/não, casos)	32/68	28/72	0,537
História de hipertensão (sim/não, casos)	26/74	30/70	0,529

IMC: índice de massa corporal; CT: colesterol total; TG: triglicerídeos; HDL-C: colesterol de lipoproteína de alta densidade; LDL-C: colesterol de lipoproteína de baixa densidade; Lp(a): lipoproteína.

### Comparação das informações gerais entre os dois grupos

O nível médio de Lp(a) no grupo sem trombose foi significativamente menor do que no grupo com trombose. Em contraste, os níveis médios de TG, IMC, pressão arterial sistólica e diastólica, glicemia de jejum, CT, HDL-C, LDL-C e os históricos de diabetes e hipertensão não apresentaram diferenças estatisticamente significativas. Esses achados fornecem um contexto valioso para a compreensão do perfil clínico geral de ambos os grupos (Tabela 1).

### Comparação dos níveis séricos de sCD40L, CCL3 e NT-ProBNP entre os grupos

A Tabela 2 apresenta a comparação dos níveis séricos de sCD40L, CCL3 e NT-ProBNP entre o grupo sem trombose (n = 100) e o grupo com trombose (n = 100). Os níveis de NT-ProBNP foram significativamente mais elevados no grupo com trombose em comparação ao grupo sem trombose. Da mesma forma, os níveis de sCD40L estavam aumentados no grupo com trombose em relação ao grupo sem trombose. Os níveis de CCL3 também apresentaram aumento significativo no grupo com trombose em comparação ao grupo sem trombose.

**Tabela 2:** Comparação dos níveis séricos de peptídeo natriurético cerebral N-terminal pró-hormonal, ligante solúvel do antígeno de diferenciação de leucócitos 40 e quimiocina ligante do motivo CC 3 entre os grupos não trombótico e trombótico (média ± DP)

Biomarcadores	Grupo sem trombose (n=100)	Grupo com trombose (n=100)	p
NT-ProBNP (pg/mL)	246,21±30,47	285,18±38,20	<0,001
sCD40L (ng/mL)	6,95±1,76	9,16±1,84	<0,001
CCL3 (ng/L)	24,16±5,36	32,29±7,58	<0,001

NT-proBNP: peptídeo natriurético cerebral N-terminal pró-hormonal; sCD40L: ligante solúvel do antígeno de diferenciação de leucócitos 40; CCL3: quimiocina ligante do motivo CC 3.

### Análise de Correlação de Pearson dos níveis de sCD40L, CCL3 e NT-ProBNP em pacientes com IC e TVMI

**Figura 1:**
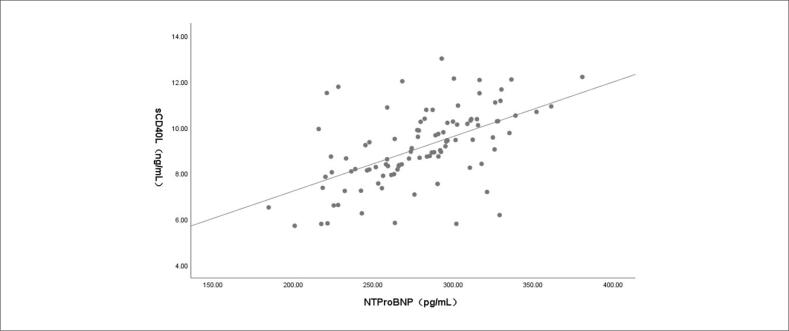
Correlação entre os níveis séricos de peptídeo natriurético cerebral N-terminal pró-hormonal (NT-proBNP) e ligante solúvel do antígeno de diferenciação de leucócitos 40 (sCD40L) em pacientes com insuficiência cardíaca e trombose venosa de membros inferiores.

**Figura 2:**
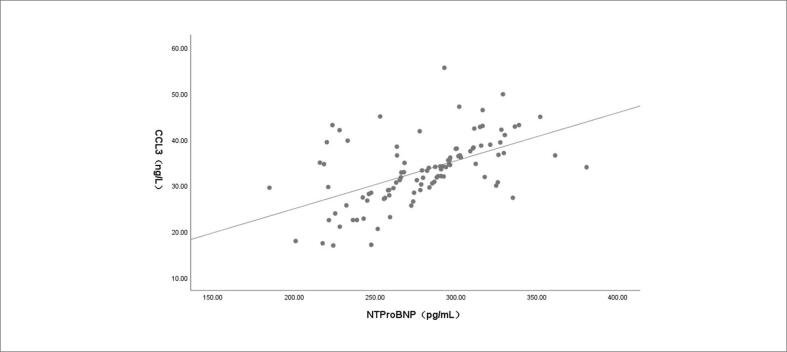
Correlação entre os níveis séricos de peptídeo natriurético cerebral N-terminal pró-hormonal (NT-proBNP) e quimiocina ligante do motivo CC 3 (CCL3) em pacientes com insuficiência cardíaca e trombose venosa de membros inferiores.

**Figura 3:**
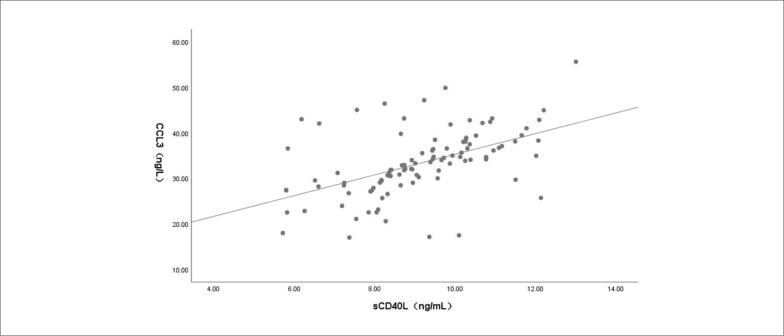
Correlação entre níveis séricos de ligante solúvel do antígeno de diferenciação de leucócitos 40 (sCD40L)4 e quimiocina ligante do motivo CC 3 (CCL3) em pacientes com insuficiência cardíaca e trombose venosa de membros inferiores.

Os resultados da análise de correlação de Pearson mostraram que, em pacientes com IC e TVMI, houve correlação positiva entre os níveis séricos de NT-ProBNP e sCD40L (r = 0,549; p < 0,001; Figura 1), entre NT-ProBNP e CCL3 (r = 0,543; p < 0,001; Figura 2) e entre sCD40L e CCL3 (r = 0,512; p < 0,001; Figura 3).

### Análise de regressão logística multivariada dos fatores que afetam o diagnóstico em pacientes com IC e TVMI

A Tabela 3 apresenta os resultados da análise de regressão logística multivariada dos fatores associados à TVMI em pacientes com IC. O NT-ProBNP aumentou significativamente as chances de trombose. Os níveis séricos de sCD40L também se destacaram como um forte preditor. Além disso, os níveis séricos de CCL3 mostraram associação significativa com o risco de trombose. Esses achados destacam sCD40L, CCL3 e NT-ProBNP como fatores críticos na avaliação do risco trombótico em pacientes com IC.

**Tabela 3:** Análise de regressão logística multivariada dos fatores que afetam a trombose venosa de membros inferiores em pacientes com insuficiência cardíaca

Variável	B	SE	Waldχ2	p	OR	IC95%
NT-ProBNP	1,834	0,704	6,784	0,009	6,257	1,574~24,867
sCD40L	0,814	0,214	14,470	<0,001	2,257	1,483~3,433
CCL3	2,105	0,931	5,113	0,024	8,208	1,324~50,900

NT-proBNP: peptídeo natriurético cerebral N-terminal pró-hormonal; sCD40L: ligante solúvel do antígeno de diferenciação de leucócitos 40; CCL3: quimiocina ligante do motivo CC 3; IC: intervalo de confiança.

Análise do valor diagnóstico dos níveis séricos de sCD40L, CCL3 e NT-ProBNP em pacientes com IC complicada por TVMI

**Figura 4:**
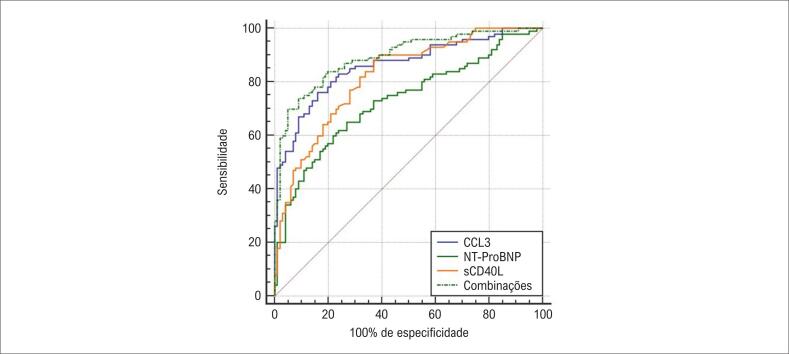
Curva ROC dos níveis séricos de peptídeo natriurético cerebral N-terminal pró-hormonal (NT-proBNP), ligante solúvel do antígeno de diferenciação de leucócitos 40 (sCD40L) e quimiocina ligante do motivo CC 3 (CCL3) no diagnóstico da insuficiência cardíaca associada à trombose venosa de membros inferiores.

A utilidade diagnóstica dos níveis séricos de sCD40L, CCL3 e NT-ProBNP em pacientes com IC e TVMI foi avaliada (Tabela 4, Figura 4). Com um valor de corte de 264,93 pg/mL, o NT-ProBNP apresentou uma AUC de 0,730, com sensibilidade de 68,00% e especificidade de 73,00%, resultando em um índice de Youden de 0,380. O nível sérico de sCD40L demonstrou desempenho diagnóstico superior, com uma AUC de 0,820 e valor de corte de 7,07 ng/mL, alcançando sensibilidade de 90,00% e especificidade de 61,00%, e um índice de Youden de 0,510. O nível sérico de CCL3 apresentou a maior acurácia diagnóstica, com uma AUC de 0,861, valor de corte de 28,96 ng/L, sensibilidade de 76,00%, especificidade de 84,00% e índice de Youden de 0,600. Quando os três biomarcadores foram combinados, a AUC aumentou para 0,894, com sensibilidade de 62,00% e especificidade de 91,00%, ressaltando o valor diagnóstico coletivo (Figura Central). A análise combinada foi superior ao desempenho diagnóstico individual de NT-ProBNP, sCD40L e CCL3 (Z para combinação vs. NT-ProBNP = 5,156, p < 0,001; Z para combinação vs. sCD40L = 3,337, p < 0,001; Z para combinação vs. CCL3 = 2,303, p = 0,021).

**Tabela 4:** Valor diagnóstico dos níveis séricos de NT-proBNP, sCD40L e CCL3 na insuficiência cardíaca associada à trombose venosa de membros inferiores

Variáveis	AUC	Ponto de corte	IC95%	Sensibilidade (%)	Especificidade (%)	Índice de Youden
NT-ProBNP	0,730	264,93 pg/mL	0,663~0,790	68,00	73,00	0,380
sCD40L	0,820	7,07 ng/mL	0,759~0,870	90,00	61,00	0,510
CCL3	0,861	28,96 ng/L	0,805~0,905	76,00	84,00	0,600
Os três indicadores combinados	0,894	-	0,843~0,933	62,00	91,00	0,650

AUC: Área sob a curva ROC; NT-proBNP: peptídeo natriurético cerebral N-terminal pró-hormonal; sCD40L: ligante solúvel do antígeno de diferenciação de leucócitos 40; CCL3: quimiocina ligante do motivo CC 3; IC: intervalo de confiança.

## Discussão

Pacientes com IC frequentemente apresentam um estado de hipercoagulabilidade, o que aumenta de forma significativa o risco de trombose. A incidência de trombose venosa em pacientes com IC crônica excede 15%, e essa taxa aumenta com o avanço da idade, maior duração da doença e piora na classificação da função cardíaca.^20^ Na prática clínica atual, pacientes com IC de alto risco são geralmente tratados com terapia anticoagulante extensa. No entanto, essa abordagem carece de evidências farmacológicas suficientes, demonstra eficácia clínica limitada e acarreta risco de reações adversas graves em pacientes com condições complexas.^21^

As reações adversas mais comuns incluem sangramentos internos, como hemorragia gastrointestinal, e sangramentos externos decorrentes de cortes ou contusões, com sintomas como hematêmese, hemoptise ou melena. Além disso, os pacientes podem desenvolver bolhas sanguíneas, equimoses e sangramento gengival, especialmente após a escovação dos dentes. O sangramento interno também pode se manifestar como cefaleias intensas ou desconforto abdominal, enquanto perdas sanguíneas significativas podem se apresentar com fraqueza ou desorientação.

Portanto, para manejar eficazmente esses riscos e garantir a segurança dos pacientes durante a terapia, os profissionais de saúde devem monitorar de perto os pacientes em uso de anticoagulantes.^22–24^ Consequentemente, o diagnóstico precoce da TVMI em pacientes com IC e o início imediato do tratamento são de vital importância.

O NT-proBNP, um neuro-hormônio essencial liberado pelas células miocárdicas do ventrículo esquerdo, é um indicador crucial da função cardíaca. O peptídeo natriurético cerebral, que promove vasodilatação e protege a função cardíaca, é produzido quando o NT-proBNP é clivado. As concentrações de NT-proBNP no sangue são modestas em condições fisiológicas normais, mas aumentam rapidamente em situações patológicas, tornando-o um marcador essencial para monitorar a função cardíaca e determinar o prognóstico em pacientes com IC.^25^

Os níveis séricos de NT-proBNP, que estão marcadamente elevados em pacientes idosos com diabetes mellitus complicado por IC com fração de ejeção preservada, foram relatados por Li et al.^26^ como um fator de risco independente. Feld et al.^27^ também identificaram uma associação significativa entre o tamanho do ventrículo esquerdo e os níveis de NT-proBNP, sugerindo que níveis elevados de NT-proBNP podem indicar maior risco de insuficiência cardiopulmonar em pacientes com doença falciforme.

Os níveis de NT-proBNP são mais altos em pacientes com fibrilação atrial do que naqueles em ritmo sinusal. A interpretação do NT-proBNP na IC é dificultada pela presença de fibrilação atrial, tornando mais complexo distinguir entre as duas condições. Além disso, comorbidades como embolia pulmonar e insuficiência renal também podem aumentar os níveis de NT-proBNP em pacientes com IC.^28^ Em indivíduos com IC crônica, os níveis de NT-proBNP são comparáveis entre homens e mulheres. No entanto, em contraste com os homens (2145 ng/L), as mulheres apresentaram um valor de corte ótimo prognóstico mais elevado (2339 ng/L). Isso sugere que valores de corte de NT-proBNP específicos para o sexo feminino podem melhorar a estratificação de risco em pacientes com IC.^29–31^

O nível sérico de NT-proBNP no grupo com trombo foi significativamente maior que no grupo sem trombo, indicando que a elevação do NT-proBNP pode contribuir para o desenvolvimento de TVMI em pacientes com IC. Além disso, a AUC para o NT-proBNP no diagnóstico de IC com TVMI foi de 0,730, sugerindo que a dosagem sérica de NT-proBNP pode servir como marcador suplementar para identificar TVMI em pacientes com IC. Para otimizar os desfechos clínicos, os médicos devem agir prontamente quando os níveis de NT-proBNP excederem 264,93 pg/mL.

Um fator conhecido como sCD40L é produzido quando as plaquetas regulam respostas inflamatórias. Após a ativação plaquetária, o sCD40L é liberado do armazenamento nos grânulos α. Os níveis plasmáticos de sCD40L são considerados influências significativas tanto na ativação plaquetária quanto no prognóstico das doenças cardiovasculares. Os níveis de sCD40L no sangue periférico mostraram-se consideravelmente mais elevados em indivíduos idosos com doença arterial coronariana e estão positivamente correlacionados com a hipertensão. Esse achado sugere que o sCD40L pode ser útil na avaliação da estabilidade e do prognóstico desses pacientes.

Além disso, Zhang et al.^32^ relataram uma associação significativa entre a reestenose vascular e os níveis séricos de sCD40L em indivíduos com doença arterial coronariana. No presente estudo, os níveis séricos de sCD40L foram significativamente mais elevados no grupo com trombo, sugerindo que o aumento de sCD40L pode estar relacionado ao desenvolvimento de TVMI em pacientes com IC. Essa observação pode ser explicada pelo estado de hipercoagulabilidade, que estimula as plaquetas e promove a liberação de sCD40L dos grânulos α para a circulação, elevando assim os níveis séricos de sCD40L.

Os resultados indicam que pacientes com IC e TVMI têm maior probabilidade de apresentar níveis sanguíneos de sCD40L > 7,07 ng/mL. A AUC diagnóstica para o sCD40L sérico na detecção de IC com TVMI foi de 0,820. Esses achados enfatizam que a determinação dos níveis séricos de sCD40L pode ser clinicamente valiosa para o diagnóstico de TVMI em pacientes idosos com IC.

O CCL3 é uma quimiocina crucial que facilita a migração de linfócitos T, mastócitos e eosinófilos, estando intimamente associado à resposta inflamatória.^33^ Além disso, pesquisas recentes demonstraram uma forte associação entre CCL3 e TVMI. Mu et al.^34^ relataram que os níveis séricos de CCL3 estavam fortemente correlacionados com TVMI pós-operatória em pacientes com câncer cervical, sugerindo que os níveis séricos de CCL3 devem ser continuamente monitorados e controlados para a prevenção e o manejo dessa condição. Os níveis séricos de CCL3 no grupo com trombo em nossa pesquisa foram consideravelmente mais elevados do que no grupo sem trombo, indicando que níveis aumentados de CCL3 podem estar associados a um maior risco de TVMI em pacientes com IC. Além disso, a AUC diagnóstica do CCL3 sérico na detecção de IC com TVMI foi de 0,861, indicando que os níveis séricos de CCL3 podem ser utilizados como marcador complementar para o diagnóstico dessa condição. Para prevenir e tratar a TVMI, os médicos devem adotar rapidamente terapia anticoagulante adequada quando os níveis sanguíneos de CCL3 ultrapassarem 28,96 ng/L.

Associações significativas entre biomarcadores foram demonstradas em pacientes com IC e TVMI por meio da análise de correlação de Pearson. Os níveis séricos de sCD40L, CCL3 e NT-proBNP apresentaram fortes correlações, sugerindo um mecanismo comum envolvendo a gravidade da IC, inflamação e trombose. Além disso, foi observada uma correlação significativa entre sCD40L e CCL3, indicando uma possível interação entre esses dois biomarcadores na promoção de processos trombóticos. Esses achados ressaltam a importância desses indicadores na avaliação do risco de trombose venosa em pacientes com IC.

Uma associação positiva foi relatada entre os níveis sanguíneos de sCD40L e CCL3 e marcadores da função de coagulação, como fibrinogênio e dímero-D, enquanto correlações negativas foram observadas com o tempo de protrombina,, tempo de trombina e tempo de tromboplastina parcial ativada. Níveis elevados de sCD40L e CCL3 também foram associados a uma maior incidência de TVP pós-operatória em indivíduos com múltiplas fraturas de costelas.^35^

Em conjunto, essas associações destacam a interação entre sCD40L, CCL3 e NT-proBNP, sugerindo que esses fatores podem atuar de forma sinérgica na indução da TVMI em pacientes com IC. Investigações adicionais são necessárias para compreender melhor essas conexões e suas implicações na estratificação de risco e na terapia individualizada nessa população de pacientes.

Um estudo de regressão logística multivariada revelou que sCD40L, CCL3 e NT-proBNP são fatores de risco significativos para o desenvolvimento de TVMI em pacientes com IC. O OR indica as associações multiplicativas entre os grupos trombótico e não trombótico (controle) para cada biomarcador associado ao risco de TVMI, com base na análise de regressão logística multivariada.

O OR de 2,257 para sCD40L indica que pacientes com níveis aumentados de sCD40L têm 2,26 vezes mais probabilidade de apresentar trombose do que o grupo controle. O CCL3 apresentou um OR de 8,208, indicando que indivíduos com níveis elevados de CCL3 têm 8,21 vezes mais probabilidade de desenvolver trombose venosa do que o grupo controle. Finalmente, um OR de 6,257 para NT-proBNP indica que pacientes com níveis aumentados de NT-proBNP têm aproximadamente 6,26 vezes mais probabilidade de apresentar trombose venosa do que o grupo controle. Esses odds ratios demonstram o risco elevado associado a cada biomarcador ao comparar os grupos trombótico e não trombótico.

A análise da curva ROC revelou que a combinação de sCD40L, CCL3 e NT-proBNP para o diagnóstico de IC com TVMI resultou em uma AUC de 0,894 e especificidade de 91,00%, superando a AUC e a especificidade individuais de cada biomarcador. Esses achados sugerem que o uso combinado de sCD40L, CCL3 e NT-proBNP aprimora a acurácia diagnóstica da IC com TVMI, oferecendo informações valiosas para o diagnóstico clínico e a terapia. Níveis elevados de sCD40L, CCL3 e NT-proBNP têm sido associados a uma maior incidência de TVMI em pacientes com IC.

Existem várias limitações no presente estudo. Primeiro, os achados podem não ser amplamente generalizáveis devido ao tamanho pequeno e homogêneo da amostra de 200 pacientes. Segundo, o desenho transversal limita a capacidade de avaliar variações temporais nos níveis de sCD40L, CCL3 e NT-proBNP. Terceiro, alguns potenciais fatores de confusão, como estilo de vida e comorbidades, podem não ter sido totalmente considerados, o que poderia ter afetado a precisão dos resultados. Quarto, o estudo não comparou fatores de risco bem estabelecidos com sCD40L, CCL3 e NT-proBNP, o que justifica investigações adicionais. Finalmente, a aplicabilidade prática dos achados pode ter sido limitada pela ausência de uma análise abrangente dos mecanismos moleculares subjacentes às correlações observadas e pela falta de avaliação de estratégias terapêuticas em relação aos níveis dos biomarcadores.

## Conclusão

Os níveis séricos de sCD40L, CCL3 e NT-proBNP foram encontrados significativamente elevados em pacientes com IC acompanhada de TVMI. O uso combinado desses biomarcadores pode facilitar o diagnóstico de TVMI em pacientes idosos com IC de forma mais rápida e precisa. São necessárias mais pesquisas experimentais para investigar os mecanismos subjacentes de sCD40L, CCL3 e NT-proBNP no contexto da IC com TVMI, uma vez que esses processos ainda são pouco compreendidos.

## Data Availability

Os conteúdos subjacentes ao texto da pesquisa estão contidos no manuscrito.
