# Supplementary material for: Valor Diagnóstico dos Níveis Séricos de sCD40L, CCL3 e NT-proBNP na Detecção da Trombose Venosa de Membros Inferiores em Pacientes Idosos com Insuficiência Cardíaca
Source: Arq Bras Cardiol. 2026 Mar 2;123(1):e20250445. [Article in Portuguese] doi: 10.36660/abc.20250445 (PMC13127813; doi:10.36660/abc.20250445)
Supplement: Translation

# Translation: Diagnostic Value of Serum sCD40L, CCL3, and NT-ProBNP Levels in Detection of Lower Limb Venous Thrombosis among Elderly Patients with Heart Failure

**DOI:** 10.36660/abc.20250445i

**Authors:** Xuelian Liu, Lina Mu, Shasha Ma, Huijing Zhang

**Affiliations:** 1 Hengshui China Department of Cardiovascular Medicine, Harrison International Peace Hospital, Hengshui – China; 2 Hengshui China Department of Surgery, Hengshui Sixth People's Hospital, Hengshui – China

**Keywords:** Trombose Venosa, Heart Failure, Aged

## Abstract

**Background:**

The serum levels of soluble CD40 ligand (sCD40L), cytokine ligand 3 (CCL3), and N-terminal pro-brain natriuretic peptide (NT-proBNP) are closely associated with heart failure (HF).

**Objective:**

This study evaluated the predictive value of serum sCD40L, CCL3, and NT-proBNP levels for lower limb venous thrombosis (LLVT) in elderly patients with HF.

**Methods:**

A total of 200 patients with HF were retrospectively included and equally divided into thrombus and non-thrombus groups based on lower limb vein ultrasound findings. Serum levels of sCD40L, CCL3, and NT-proBNP were measured and analyzed. A p-value < 0.05 was considered statistically significant.

**Results:**

Serum sCD40L, CCL3, and NT-proBNP levels were significantly higher in the thrombus group compared with the non-thrombus group (p < 0.05). Multivariate logistic regression analysis demonstrated that elevated serum sCD40L, CCL3, and NT-proBNP levels are risk factors for LLVT in patients with HF (p < 0.05). The area under the receiver operating characteristic (ROC) curve (AUC) for the combined serum sCD40L, CCL3, and NT-proBNP levels was greater than that of each individual marker (Z values: sCD40L = 3.337, CCL3 = 2.303, and NT-proBNP = 5.156; p < 0.001, p < 0.001, and p = 0.021, respectively).

**Conclusion:**

Elevated serum sCD40L, CCL3, and NT-proBNP levels were associated with LLVT in elderly patients with HF. The combination of these markers provides high diagnostic value for the detection of LLVT in this population.

## Introduction

Heart failure (HF) is a multifactorial clinical disease that results from structural or functional defects of the heart, limiting its ability to contract or the ventricles to relax. The incidence of HF in China has been steadily increasing in parallel with population aging.^1,2^ Patients with HF typically present with symptoms such as dyspnea, fatigue, and fluid retention.^1,2^

Lower limb venous thrombosis (LLVT) is a common concomitant disorder in HF patients. Venous thromboembolism (VTE), which encompasses both pulmonary embolism (PE) and deep vein thrombosis (DVT/LLVT), poses a particularly serious risk for individuals with HF. Indeed, HF patients have a 2–3 times higher risk of developing LLVT and VTE compared to the general population.^3,4^ This increased risk is largely attributed to factors such as immobility, acute infections, and comorbidities including chronic obstructive pulmonary disease, which itself elevates the risk of VTE.^5–7^ HF patients may experience severe consequences from DVT, including right ventricular failure and increased mortality. Reduced blood flow velocity in the lower limbs due to DVT leads to venous wall inflammation and a hypercoagulable state, which impairs venous return. Mortality risk may rise if the thrombus dislodges and causes a pulmonary embolism.^8,9^ Therefore, accurate diagnosis and timely intervention in HF patients with LLVT are essential to improving prognosis.

A DVT in HF patients is associated with risk factors, such as advanced age, female gender, and a history of DVT or PE. Risk factors include inherited coagulation abnormalities, long-term illnesses (such as cancer), obesity (a high body mass index [BMI]), and extended immobility. Additional factors that may raise vulnerability include central venous catheters, hormonal effects (such as estrogen from hormone therapy), and injury to the veins from trauma or surgery. Taken together, these variables demonstrate the intricate interaction between clinical and demographic factors that influence the risk of a DVT in patients with HF.^10,11^

**Figure:** 1. Serum sCD40L, CCL3, and NT-proBNP levels were significantly higher in the thrombus group compared with the non-thrombus group; 2. Elevated serum sCD40L, CCL3, and NT-proBNP levels represent risk factors for LLVT in patients with HF; 3. The area under the receiver operating characteristic (ROC) curve for the combined serum sCD40L, CCL3, and NT-proBNP levels was greater than that of each individual marker.

The degree and prognosis of HF are correlated with the biomarker N-terminal pro-brain natriuretic peptide (NT-ProBNP), which reflects cardiac function.^12^ NT-ProBNP is essential for diagnosing and evaluating HF. Studies have shown that inflammatory factors have an important role in the formation of a DVT.^13^ To regulate inflammatory responses, platelets release soluble leukocyte differentiation antigen 40 ligand (sCD40L), which is a transmembrane glycoprotein that is a member of the tumor necrosis factor receptor family.^14^ Jin et al.^15^ reported that elderly patients with severe HF have elevated serum sCD40L levels. The interaction between CD40 and sCD40L is critical in inflammatory conditions. The sCD40L level is much greater in HF patients, especially in patients with severe HF, diabetes, or hypertension than healthy controls. An increased sCD40L level may have an impact on vascular function and cardiac remodeling, as evidenced by the correlation with clinical severity, neurohormonal imbalance, and left ventricular dysfunction.^16^ Another report showed that patients with bronchial asthma and patients with osteoporosis have elevated levels of the pro-inflammatory chemokine, CCL3, which is important in controlling inflammatory processes.^17^

Venous ultrasound of the lower extremities is considered the "gold standard" for diagnosing DVT; however, it is a high-cost procedure. Serum biomarkers can be used clinically to identify indices associated with DVT and may facilitate diagnosis in elderly patients with HF. The diagnostic application of serum sCD40L, CCL3, and NT-proBNP levels in HF patients with DVT has been limited. The present study evaluated the diagnostic utility of serum sCD40L, CCL3, and NT-proBNP levels in 200 elderly HF patients, aiming to provide guidance for the clinical diagnosis of HF with concomitant DVT.

## Materials and methods

A total of 200 elderly patients with HF who received treatment at our institution between July 2020 and July 2022 were enrolled in the current study. Patients were categorized into two groups (thrombus and non-thrombus) based on the results of lower leg venous ultrasonography at the time of admission. This study adhered to the ethical principles and standards for human experimentation and was approved by the Medical Ethics Committee of our hospital (No. 2022-1-035). Each participant gave written informed consent.

### Inclusion criteria

The inclusion criteria were as follows: Diagnosis of HF based on the 2021 Expert Consensus on the Quality Evaluation and Control Indicators for the Diagnosis and Treatment of Heart Failure in China, considering medical history, symptoms, signs, echocardiography, and cardiac function assessment;^18,19^ agreement to undergo lower limb venous ultrasonography; age greater than 60 years; availability of complete clinical data.

### Exclusion criteria

The exclusion criteria were as follows: Presence of lower limb varicose veins, malignancies, hematologic diseases, PE, diabetes, atrial fibrillation, or COPD; use of anticoagulants (including warfarin, rivaroxaban, dabigatran, or low-molecular-weight heparin) prior to admission; pregnancy or lactation; severe dysfunction of vital organs, including the heart, liver, spleen, or kidneys; lack of informed consent from the patient or the patient's family.

### Main reagents and instruments

The following instruments and reagents were used in this study: Philips Epiq 5C (Philips Healthcare, City, the Netherlands); Hitachi 7600 Automated Biochemical Analyzer (Hitachi High-Technologies Corporation, City, Japan); FK-SY96S Multifunctional Microplate Reader (Shandong Lain Optoelectronics Technology Co., Ltd., City, China); and sCD40L and CCL3 ELISA kits (Abcam, Cambridge, UK).

### Collection of general information

Data were collected from all participants, including age, sex, BMI, systolic blood pressure, diastolic blood pressure, fasting blood glucose, total cholesterol (TC), triglycerides (TG), high-density lipoprotein cholesterol (HDL-C), low-density lipoprotein cholesterol (LDL-C), lipoprotein (a) [Lp(a)], and medical history of diabetes and hypertension, along with other clinical indicators.

### Ultrasound examination

A technician performed bedside lower limb venous ultrasound examinations immediately after participants were enrolled in the study using an ultrasound machine equipped with a high-frequency linear array probe (probe frequency range, 6–12 MHz. The assessment included the common femoral vein, femoral vein, deep femoral vein (profunda femoris vein), popliteal vein, anterior tibial vein, posterior tibial vein, peroneal vein, dorsal vein of the foot, intermuscular veins of the lower leg, and communicating veins. The diagnosis of LLVT was based on the criteria outlined in the 2018 publication, *Lower Limb Venous Ultrasound: A Review of the American Institute of Ultrasound in Medicine Guidelines for Peripheral Venous Ultrasound Examination*.^19^ Patients were subsequently classified into thrombus or non-thrombus groups.

### Serum collection and storage

On the morning following admission, 4 mL of peripheral blood was collected from each HF patient into tubes and left to stand at room temperature for 30 minutes. To obtain serum, the samples were centrifuged for 3 minutes at 3000 rpm. The serum was then aliquoted into sterile EP tubes and stored at −20 °C.

### Measurement of Serum sCD40L, CCL3, and NT-ProBNP Levels

Serum NT-ProBNP levels were measured using an automated biochemical analyzer. Serum concentrations of sCD40L and CCL3 were determined according to the manufacturer's instructions provided with the respective assay kits.

### Statistical analysis

Statistical analyses were performed using SPSS version 23.0. Continuous variables are presented as mean ± standard deviation (SD), and normality was assessed using the Shapiro–Wilk test. Differences in baseline characteristics and in sCD40L, CCL3, and NT-ProBNP levels between the two groups were compared using the unpaired Student's t-test. Associations among sCD40L, CCL3, and NT-ProBNP levels were evaluated using Pearson correlation analysis. The occurrence of LLVT in patients with HF was investigated using logistic regression analysis. Independent variables included Lp(a), platelet count, creatine kinase, sCD40L, CCL3, and NT-ProBNP levels in the multivariate logistic regression model. The diagnostic performance of serum sCD40L, CCL3, and NT-ProBNP levels for identifying venous thrombosis in HF was assessed using receiver operating characteristic (ROC) curve analysis. Comparisons of the area under the ROC curve (AUC) were performed using the Z-test. A P value < 0.05 was considered statistically significant.

## Results

General clinical data were collected from 200 patients, who were divided into a non-thrombosis group (n = 100) and a thrombosis group (n = 100). There were no significant differences in sex or age between the two groups (Table 1).

**Table 1:** Comparison of general clinical data between the non-thrombosis and thrombosis groups (Mean±SD)

Clinical indicators	Non-thrombosis Group (n=100)	Thrombosis Group (n=100)	p
Age (years)	69.12±5.26	68.54±5.84	0.461
Male/female (cases)	50/50	55/45	0.479
BMI (kg/m^2^)	22.18±2.69	21.92±3.02	0.521
Systolic blood pressure (mmHg)	132.54±17.48	136.20±18.17	0.148
Diastolic blood pressure (mmHg)	81.25±9.62	83.33±9.34	0.122
Fasting blood glucose (mmol/L)	4.86±0.85	4.93±0.74	0.535
TC (mmol/L)	4.05±0.36	4.09±0.40	0.458
TG (mmol/L)	2.26±0.18	2.31±0.20	0.065
HDL-C (mmol/L)	1.89±0.52	1.95±0.47	0.393
LDL-C (mmol/L)	2.65±0.30	2.68±0.32	0.495
Lp(a) (mg/L)	187.56±26.28	332.04±37.41	<0.001
History of diabetes mellitus (yes/no, cases)	32/68	28/72	0.537
History of hypertension (yes/no, case)	26/74	30/70	0.529

BMI: body mass index; TC: total cholesterol; TG: triglycerides; HDL-C: high-density lipoprotein-cholesterol; LDL-C: low-density lipoprotein-cholesterol. Lp(a): lipoprotein.

### Comparison of general information between the two groups

The average Lp(a) level in the non-thrombosis group was significantly lower than that in the thrombosis group. In contrast, average TG levels, BMI, systolic and diastolic blood pressure, fasting blood glucose, TC, HDL-C, LDL-C, and histories of diabetes and hypertension showed no statistically significant differences. These findings provide valuable context for understanding the overall clinical profile of both groups (Table 1).

### Comparison of Serum sCD40L, CCL3, and NT-ProBNP levels between groups

Table 2 presents the comparison of serum sCD40L, CCL3, and NT-ProBNP levels between the non-thrombotic group (n = 100) and the thrombotic group (n = 100). NT-ProBNP levels were significantly higher in the thrombotic group than in the non-thrombotic group. Similarly, sCD40L levels were elevated in the thrombotic group compared with the non-thrombotic group. CCL3 levels were also significantly increased in the thrombotic group relative to the non-thrombotic group.

**Table 2:** Comparison of serum N-terminal pro-brain natriuretic peptide, soluble leukocyte differentiation antigen 40 ligand and chemokine CC motif ligand 3 levels between the non-thrombotic and thrombotic groups (mean ± SD)

Biomarkers	Non-thrombotic group (n=100)	Thrombosis group (n=100)	p
NT-ProBNP (pg/mL)	246.21±30.47	285.18±38.20	<0.001
sCD40L (ng/mL)	6.95±1.76	9.16±1.84	<0.001
CCL3 (ng/L)	24.16±5.36	32.29±7.58	<0.001

NT-ProBNP: N-terminal pro-brain natriuretic peptide; sCD40L: soluble leukocyte differentiation antigen 40 ligand; CCL3: chemokine CC motif ligand 3.

### Pearson correlation analysis of sCD40L, CCL3, and NT-ProBNP Levels in HF patients with LLVT

The results of the Pearson correlation analysis showed that in HF patients with LLVT, there was a positive correlation between serum NT-ProBNP and sCD40L levels (r = 0.549; p < 0.001; Figure 1), between NT-ProBNP and CCL3 levels (r = 0.543; p < 0.001; Figure 2), and between sCD40L and CCL3 levels (r = 0.512; p < 0.001; Figure 3).

**Figure 1:** Correlation between serum N-terminal pro-brain natriuretic peptide (NT-ProBNP) and soluble leukocyte differentiation antigen 40 ligand (sCD40L) levels in patients with heart failure and lower extremity venous thrombosis.

**Figure 2:** Correlation between serum N-terminal pro-brain natriuretic peptide (NT-ProBNP) and chemokine CC motif ligand 3 (CCL3) levels in patients with heart failure and lower extremity venous thrombosis.

**Figure 3:** Correlation between serum soluble leukocyte differentiation antigen 40 ligand (sCD40L) and chemokine CC motif ligand 3 (CCL3) levels in patients with heart failure and lower extremity venous thrombosis.

### Multivariate logistic regression analysis of factors affecting aiagnosis in HF patients with LLVT

Table 3 shows the multivariate logistic regression analysis of factors influencing Table 3 presents the results of the multivariate logistic regression analysis of factors associated with LLVT in HF patients. NT-ProBNP significantly increased the odds of thrombosis. Serum sCD40L levels also emerged as a strong predictor. Additionally, serum CCL3 levels were significantly associated with thrombosis risk. These findings highlight sCD40L, CCL3, and NT-ProBNP as critical factors in assessing thrombotic risk among patients with HF.

**Table 3:** Multivariate logistic regression analysis of factors influencing lower extremity venous thrombosis in patients with heart failure

Variable	B	SE	Waldχ2	p	OR	95%CI
NT-ProBNP	1.834	0.704	6.784	0.009	6.257	1.574~24.867
sCD40L	0.814	0.214	14.470	<0.001	2.257	1.483~3.433
CCL3	2.105	0.931	5.113	0.024	8.208	1.324~50.900

NT-ProBNP: N-terminal pro-brain natriuretic peptide; sCD40L: soluble leukocyte differentiation antigen 40 ligand; CCL3: chemokine CC motif ligand 3; CI: confidence interval.

### Diagnostic value analysis of serum sCD40L, CCL3, and NT-ProBNP levels for HF complicated by LLVT

The diagnostic utility of serum sCD40L, CCL3, and NT-ProBNP levels in patients with HF and LLVT was evaluated (Table 4, Figure 4). With a cut-off value of 264.93 pg/mL, NT-ProBNP yielded an AUC of 0.730, with a sensitivity of 68.00% and specificity of 73.00%, resulting in a Youden index of 0.380. Serum sCD40L demonstrated superior diagnostic performance, with an AUC of 0.820 and a cut-off value of 7.07 ng/mL, achieving a sensitivity of 90.00% and specificity of 61.00%, and a Youden index of 0.510. Serum CCL3 showed the highest diagnostic accuracy, with an AUC of 0.861, a cut-off value of 28.96 ng/L, sensitivity of 76.00%, specificity of 84.00%, and a Youden index of 0.600. When all three biomarkers were combined, the AUC increased to 0.894, with a sensitivity of 62.00% and specificity of 91.00%, underscoring their collective diagnostic value (Central Figure). The combined analysis was superior to the individual diagnostic performance of NT-ProBNP, sCD40L, and CCL3 (Z for combination vs. NT-ProBNP = 5.156, p < 0.001; Z for combination vs. sCD40L = 3.337, p < 0.001; Z for combination vs. CCL3 = 2.303, p = 0.021).

**Figure 4:** ROC curve for serum N-terminal pro-brain natriuretic peptide (NT-ProBNP), serum soluble leukocyte differentiation antigen 40 ligand (sCD40L) and chemokine CC motif ligand 3 (CCL3) levels in diagnosing heart failure with lower extremity venous thrombosis.

**Table 4:** Diagnostic value of serum NT-ProBNP, sCD40L, and CCL3 levels in heart failure combined with lower extremity venous thrombosis

Variables	AUC	Cut-off	95%CI	Sensitivity (%)	Specificity (%)	Youden index
NT-ProBNP	0.730	264.93 pg/mL	0.663~0.790	68.00	73.00	0.380
sCD40L	0.820	7.07 ng/mL	0.759~0.870	90.00	61.00	0.510
CCL3	0.861	28.96 ng/L	0.805~0.905	76.00	84.00	0.600
All three indicators combined	0.894	-	0.843~0.933	62.00	91.00	0.650

AUC: Area under the ROC curve; NT-ProBNP: N-terminal pro-brain natriuretic peptide; sCD40L: soluble leukocyte differentiation antigen 40 ligand; CCL3: chemokine CC motif ligand 3; CI: confidence interval.

## Discussion

Patients with HF often present with a hypercoagulable state, which markedly increases the risk of thrombosis. The incidence of venous thrombosis in patients with chronic HF exceeds 15% and this rate rises with advancing age, longer disease duration, and worsening cardiac function classification.^20^ In current clinical practice, high-risk HF patients are generally treated with extensive anticoagulation therapy. However, this approach lacks sufficient pharmacological evidence, demonstrates limited clinical effectiveness, and carries the risk of severe adverse reactions in patients with complex conditions.^21^

Common adverse reactions include internal bleeding, such as gastrointestinal hemorrhage, and external bleeding from cuts or bruises, with symptoms such as hematemesis, hemoptysis, or melena. In addition, patients may develop blood blisters, bruises, and bleeding gums, particularly after tooth brushing. Internal bleeding may also manifest as severe headaches or abdominal discomfort, while significant blood loss may present with weakness or disorientation.

Therefore, to effectively manage these risks and ensure patient safety during therapy, healthcare practitioners must closely monitor patients receiving anticoagulants.^22–24^ Consequently, early diagnosis of LLVT in HF patients and prompt initiation of treatment are vitally important.

NT-ProBNT, an essential neurohormone released by left ventricular myocardial cells, is a crucial indicator of heart function. Brain natriuretic peptide, which promotes vasodilation and safeguards heart function, is produced when NT-ProBNP is cleaved. NT-ProBNP concentrations in the blood are modest in normal physiological conditions, but rise rapidly in pathological situations, making it an essential marker for monitoring cardiac function and determining prognosis in patients with HF.^25^

Serum NT-ProBNP levels, which are markedly elevated in older patients with diabetes mellitus complicated by HF with preserved ejection fraction, have reported by Li et al.^26^ as an independent risk factor. Feld et al.^27^ also identified a significant association between left ventricular size and NT-proBNP levels, suggesting that elevated NT-proBNP may indicate a higher risk of cardiopulmonary failure in patients with sickle cell disease.

NT-proBNP levels are higher in patients with atrial fibrillation than in those in sinus rhythm. The interpretation of NT-proBNP in HF is complicated by the presence of AF, making it more difficult to distinguish between the two conditions. Furthermore, comorbidities such as pulmonary embolism and renal failure may also increase NT-proBNP levels in HF patients.^28^ In individuals with chronic HF, NT-proBNP levels are comparable between men and women. In contrast to males (2145 ng/L), women had a higher NT-proBNP optimum prognostic cut-off (2339 ng/L). This implies that NT-proBNP cut-offs tailored to female gender might enhance risk classification for HF patients.^29–31^

The serum NT-proBNP level in the thrombus group was significantly higher than in the non-thrombus group, indicating that elevated NT-proBNP may contribute to LLVT development in HF patients. Additionally, the AUC for NT-proBNP in diagnosing HF with LLVT was 0.730, suggesting that serum NT-proBNP measurement could serve as a supplementary marker for identifying LLVT in HF. To optimize patient outcomes, physicians should act promptly when NT-proBNP levels exceed 264.93 pg/mL.

A factor known as sCD40L is produced when platelets regulate inflammatory responses. Upon platelet activation, sCD40L is released from storage in α-granules. Plasma levels of sCD40L are thought to significantly influence both platelet activation and the prognosis of cardiovascular disease. Peripheral blood sCD40L levels have been shown to be considerably higher in older individuals with coronary artery disease and are positively correlated with hypertension. This finding suggests that sCD40L may be useful in assessing disease stability and prognosis in these patients.

Additionally, Zhang et al.^32^ reported a significant association between vascular restenosis and serum sCD40L levels in individuals with coronary artery disease. In the present study, serum sCD40L levels were significantly higher in the thrombus group, suggesting that elevated sCD40L may be linked to the development of LLVT in patients with HF. This observation may be explained by the hypercoagulable state, which stimulates platelets and promotes the release of sCD40L from α-granules into the circulation, thereby increasing serum sCD40L levels.

The results indicate that patients with HF and LLVT are more likely to have blood sCD40L levels > 7.07 ng/mL. The diagnostic AUC for serum sCD40L in detecting HF with LLVT was 0.820. These findings emphasize that measuring serum sCD40L levels may be clinically valuable for diagnosing LLVT in elderly patients with HF.

CCL3 is a crucial chemokine that facilitates the migration of T lymphocytes, mast cells, and eosinophils and is closely linked to the inflammatory response.^33^ Additionally, recent research has demonstrated a robust association between CCL3 and LLVT. Serum CCL3 levels were reported to be strongly correlated with postoperative LLVT in patients with cervical cancer by Mu et al.,^34^ suggesting that serum CCL3 levels should be continuously monitored and controlled for the prevention and management of this disease. Serum CCL3 levels in the thrombus group of our research were considerably higher than the non-thrombus group, indicating that elevated CCL3 levels could be associated with a higher risk of LLVT in patients with HF. Furthermore, the serum CCL3 diagnostic AUC in detecting HF with LLVT was 0.861, indicating that serum CCL3 levels may be used as a backup sign for the diagnosis of this illness. To prevent and cure LLVT, physicians should rapidly adopt adequate anticoagulant medication when the blood CCL3 levels surpasses 28.96 ng/L.

Significant associations among biomarkers were demonstrated in HF patients with LLVT using Pearson correlation analysis. Serum sCD40L, CCL3, and NT-ProBNP levels showed strong correlations, suggesting a common mechanism involving HF severity, inflammation, and thrombosis. Furthermore, a significant correlation was observed between sCD40L and CCL3, indicating a possible interaction between these two biomarkers in promoting thrombotic processes. These findings underscore the importance of these indicators in assessing the risk of venous thrombosis in HF patients.

A positive association has been reported between blood sCD40L and CCL3 levels and coagulation function markers such as fibrinogen (FIB) and D-dimer, while negative correlations were observed with prothrombin time, thrombin time, and activated partial thromboplastin time. Elevated levels of sCD40L and CCL3 have also been linked to an increased incidence of postoperative DVT in individuals with multiple rib fractures.^35^

Taken together, these associations highlight the interplay among sCD40L, CCL3, and NT-proBNP, suggesting that these factors may act synergistically to drive LLVT in HF patients. Further investigation is warranted to better understand these connections and their implications for risk stratification and individualized therapy in this patient population.

A multivariate logistic regression study revealed that sCD40L, CCL3, and NT-ProBNP are significant risk factors for the development of LLVT in HF patients. The OR indicates the multiplicative associations between the thrombotic and non-thrombotic (control) groups for each biomarker associated with the risk of LLVT based on multivariate logistic regression analysis.

The OR of 2.257 for sCD40L indicates that patients with increased sCD40L levels are 2.26 times more likely to have thrombosis than the control group. CCL3 had an OR of 8.208, indicating that people with increased CCL3 levels are 8.21 times more likely to have venous thrombosis than the control group. Finally, an OR of 6.257 for NT-ProBNP indicates that patients with increased NT-ProBNP levels are around 6.26 times more likely to have venous thrombosis than the control group. These odds ratios demonstrate the elevated risk associated with each biomarker when comparing the thrombotic and non-thrombotic groups.

The ROC curve analysis revealed that combining sCD40L, CCL3, and NT-proBNP for diagnosing HF with LLVT resulted in an AUC of 0.894 and a specificity of 91.00%, surpassing the individual AUC and specificity of each biomarker. These findings suggest that the combined use of sCD40L, CCL3, and NT-proBNP enhances diagnostic accuracy for HF with LLVT, offering valuable insights for clinical diagnosis and therapy. Elevated levels of sCD40L, CCL3, and NT-proBNP have been associated with an increased incidence of LLVT in HF patients.

There are several limitations to the current study. First, the findings may not be broadly generalizable due to the small and homogeneous sample size of 200 patients. Second, the cross-sectional design limits the ability to evaluate temporal variations in sCD40L, CCL3, and NT-proBNP levels. Third, some potential confounding variables, such as lifestyle factors and comorbidities, may not have been fully accounted for, which could have affected the accuracy of the results. Fourth, the study did not compare well-established risk factors with sCD40L, CCL3, and NT-proBNP, warranting further investigation. Finally, the practical applicability of the findings may have been constrained by the lack of a comprehensive examination of the molecular mechanisms underlying the observed correlations and the absence of an assessment of treatment strategies in relation to biomarker levels.

## Conclusion

Serum levels of sCD40L, CCL3, and NT-proBNP were found to be markedly elevated in patients with HF accompanied by LLVT. The combined use of these biomarkers may facilitate the diagnosis of LLVT in elderly patients with HF more rapidly and accurately. Further experimental research is warranted to investigate the underlying mechanisms of sCD40L, CCL3, and NT-proBNP in the context of HF with LLVT, as these processes remain poorly understood.

## Data Availability

The underlying content of the research text is contained within the manuscript.
